# Patterns of host–parasite associations in tropical lice and their passerine hosts in Cameroon

**DOI:** 10.1002/ece3.6386

**Published:** 2020-06-18

**Authors:** Magdalena Gajdošová, Oldřich Sychra, Jakub Kreisinger, Ondřej Sedláček, Eric Djomo Nana, Tomáš Albrecht, Pavel Munclinger

**Affiliations:** ^1^ Department of Zoology Faculty of Science Charles University Prague Czech Republic; ^2^ Department of Ecology Faculty of Science Charles University Prague Czech Republic; ^3^ Department of Biology and Wildlife Diseases Faculty of Veterinary Hygiene and Ecology University of Veterinary and Pharmaceutical Sciences Brno Czech Republic; ^4^ Institute of Agricultural Research for Development (IRAD) Messa ‐Yaoundé Cameroon; ^5^ Institute of Vertebrate Biology Czech Academy of Sciences Brno Czech Republic

**Keywords:** cospeciation, feather lice, host switching, host–parasite associations, passerines, tropical ecology

## Abstract

Coevolutionary processes that drive the patterns of host–parasite associations can be deduced through congruence analysis of their phylogenies. Feather lice and their avian hosts have previously been used as typical model systems for congruence analysis; however, such analyses are strongly biased toward nonpasserine hosts in the temperate zone. Further, in the Afrotropical region especially, cospeciation studies of lice and birds are entirely missing. This work supplements knowledge of host–parasite associations in lice using cospeciation analysis of feather lice (genus *Myrsidea* and the *Brueelia* complex) and their avian hosts in the tropical rainforests of Cameroon. Our analysis revealed a limited number of cospeciation events in both parasite groups. The parasite–host associations in both louse groups were predominantly shaped by host switching. Despite a general dissimilarity in phylogeny for the parasites and hosts, we found significant congruence in host–parasite distance matrices, mainly driven by associations between *Brueelia* lice and passerine species of the Waxbill (Estrildidae) family, and *Myrsidea* lice and their Bulbul (Pycnonotidae) host species. As such, our study supports the importance of complex biotic interactions in tropical environments.

## Graphical abstract

Associations of lice and their avian hosts in the tropical rainforests of Cameroon are predominantly shaped by host switching. Despite the general incongruence between parasite and host phylogenies, the significant correlation between host and parasite phylogenetic distances suggests the prevalence of host switching to closely related hosts.

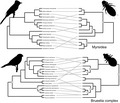

## 1 INTRODUCTION

Resolving the processes that drive the patterns of host–parasite associations is an essential goal of evolutionary parasitology and could contribute to our understanding of parasite distribution and biodiversity. New associations may be established following cospeciation, when host‐specific parasites speciate as a response to speciation of the host. If cospeciation events represent the prevailing source of new host–parasite interactions, the parasite phylogeny should mirror that of the host with respect to both topology and age of the nodes, referred to as Fahrenholz's rule (Eichler, 1948; Farenholz, 1913). On the other hand, parasites may also colonize new hosts via horizontal host switching, which may lead to incongruence in parasite and host phylogenies. While there are a number of potential sources of tree incongruence, for example, sorting events, including parasite extinction, duplication (intrahost speciation), and cohesion (failure to speciate), comparisons of host and parasite phylogenies can be used as a cue for revealing the role of cospeciation and host switching in a given host–parasite system (Page, 2003).

Feather lice represent a convenient, repeatedly used model for cospeciation studies as they are regularly host specific, their entire life cycle takes place on the body of a single host, their survival outside the host is limited, and they are predominantly transmitted vertically between parents and offspring (Price, Hellenthal, Palma, Johnson, & Clayton, 2003). Cospeciation analysis has frequently been applied to feather lice and their avian hosts (de Vienne et al., 2013; Table 1), the results indicating a wide spectrum of potential processes that drive the patterns of host–parasite associations. While incongruences between phylogenies of some feather lice and their hosts suggest that host–parasite associations were mainly established through host switching (e.g., Banks, Palma, & Paterson, 2006; Johnson, Adams, & Clayton, 2002; Weckstein, 2004), phylogenies of other louse groups strongly mirror the phylogenies of their hosts and hence advocate a predominant role for cospeciation (e.g., Page et al., 2004; Paterson, Wallis, Wallis, & Gray, 2000). In addition to differences in the methodological approaches used in cospeciation studies, various parasite species’ life‐history traits may affect the ratio between cospeciation and host switching during the formation of host–parasite associations (Clayton, Bush, & Johnson, 2004). For example, while parasite physiological adaptations to the host apparently support cospeciation (Clayton, Bush, Goates, & Johnson, 2003), phoresis (mechanical transport by louse flies) favors host switching (e.g., Harbison & Clayton, 2011; Johnson et al., 2002). On the other hand, host life‐history traits may affect the frequency and pattern of host switching. According to the “resource tracking hypothesis,” a parasite should switch to a new host on which it can continue to exploit the same resources (Timm, 1983). Exploitation of the new host may be thwarted, however, by a difference between the former and new host that increases with their phylogenetic distance (Engelstädter & Hurst, 2006). The importance of host relatedness has been demonstrated by "natural" experiments, in which lice fail to establish on brood parasites (e.g., cuckoos and indigobirds) despite close contact between the young brood parasites and foster parents in the nest (Balakrishnan & Sorenson, 2007; Brooke & Nakamura, 1998). Difference in body temperature, feather structure, or host immune and behavioral defenses may considerably lower parasite fitness, such that a host switch would result in an evolutionary dead end. Indeed, transfer experiments have shown that lice find it difficult to survive on alien host species (Clayton, Bush, et al., 2003; Tompkins & Clayton, 1999). On the other hand, as lice are parasites with limited dispersal ability, patterns of host shifting will be greatly affected simply by the probability of encountering new hosts (Clayton et al., 2004).

**TABLE 1:** Cospeciation analysis of feather lice and their avian hosts

Parasite	Host	Host speciations accompanied by parasite cospeciation	Significant amount of cospeciation events or phylogenetic congruence	Source
*Alcedoecus* (Ischnocera: Philopteridae)	Halcyoninae (Coraciiiformes)	4 of 5 (80%)		Catanach et al. (2019)
*Alcedofulla* (Ischnocera: Philopteridae)	Alcedininae (Coraciiformes)	5 of 8 (62.5%)		Catanach et al. (2019)
*Alcedofulla* (Ischnocera: Philopteridae)	Cerylinae (Coraciiformes)	4 of 6 (66.6%)	†	Catanach et al. (2019)
*Auricotes*, *Campanulotes*, *Coloceras, Physconelloides* (Ischnocera: Philopteridae)	Columbiformes	7 of 19 (36.8%)	†	Johnson and Clayton (2003)
*Auricotes*, *Campanulotes*, *Coloceras, Physconelloides* (Ischnocera: Philopteridae)	Columbiformes	22 of 51 (43.1%)	†	Sweet, Boyd, and Johnson (2016)
*Austrogoniodes* (Ischnocera: Philopteridae)	Sphenisciformes	4 of 17 (23.5%)		Banks et al. (2006)
Subspecies of *Austrophilopterus cancellosus* (Ischnocera: Philopteridae)	*Ramphastos* toucans (Piciformes)	1 of 10 (10%)		Weckstein (2004)
*Paraclisis* (Ischnocera: Philopteridae)	Procellariiformes	9 of 11 (81.8%)	†	Page et al. (2004)
*Brueelia s.l.* (Ischnocera: Philopteridae)	Several orders, mainly Passeriformes	5 of 24 (20.8%)		Johnson et al. (2002)
*Brueelia s.l.* (Ischnocera: Philopteridae)	Passeriformes	NA	†	Sweet et al. (2018)
*Coloceras, Campanulotes, Physconelloides* (Ischnocera: Philopteridae)	Columbiformes	3 of 11 (27.3%)		Sweet et al. (2017)
*Columbicola* (Ischnocera: Philopteridae)	Columbiformes	7 of 19 (36.8%)	†	Johnson and Clayton (2003)
*Columbicola* (Ischnocera: Philopteridae)	Columbiformes	3 of 12 (25%)		Clayton and Johnson (2003)
*Columbicola* (Ischnocera: Philopteridae)	Columbiformes	7 of 22 (31.8%)	†	Clayton, Bush, et al. (2003)
*Columbicola* (Ischnocera: Philopteridae)	Columbiformes	7 of 27 (25.9%)	†	Johnson, Adams, Page, and Clayton (2003)
*Columbicola* (Ischnocera: Philopteridae)	Columbiformes	14 of 51 (27.4%)	†	Sweet et al. (2016)
*Columbicola* (Ischnocera: Philopteridae)	Columbiformes	1 of 12 (8.3%)	†	Sweet and Johnson (2016)
*Columbicola* (Ischnocera: Philopteridae)	Columbiformes	8 of 11 (72.7%)	†	Sweet et al. (2017)
*Columbicola* (Ischnocera: Philopteridae)	Columbiformes	1 of 12 (8.3%)	†	Sweet and Johnson (2018)
*Docophoroides* (Ischnocera: Philopteridae)	Procellariiformes	5 of 8 (62.5%)		Page et al. (2004)
*Episbates*, *Perineus*, *Harrisoniella* (Ischnocera: Philopteridae)	Procellariiformes	6 of 10 (60%)		Page et al. (2004)
*Halipeurus* (Ischnocera: Philopteridae)	Procellariiformes	4 of 4 (100%)	†	Paterson and Banks (2001)
*Halipeurus* (Ischnocera: Philopteridae)	Procellariiformes	6 of 12 (50%)		Page et al. (2004)
*Halipeurus* (Ischncoera: Philopteridae)	Procellariiformes	†		Hammer, Brown, Bugoni, Palma, and Hughes (2010)
*Paraclisis* (Ischnocera: Philopteridae)	Procellariiformes	9 of 11 (81.8%)	†	Page et al. (2004)
*Pectinopygus* (Ischnocera: Philopteridae)	Pelecaniformes	10–12 of 17 (59%–71%)	†	Hughes, Kennedy, Johnson, Palma, and Page (2007)
Philopteridae (Ischnocera)	Procellariiformes and Sphenisciformes		†	Paterson and Gray (1997)
Philopteridae (Ischnocera)	Procellariiformes and Sphenisciformes	9 of 10 (90%)	†	Paterson et al. (2000)
Philopteridae (Ischnocera)	aquatic birds	5 of 9 (55.5%)		Johnson, Kennedy, and Mccracken (2006)
*Physconelloides* (Ischnocera: Philopteroides)	Columbiformes	8 of 12 (66.7%)	†	Clayton and Johnson (2003)
Philopteridae (Ischnocera)	Many bird orders	6 of 36 (16.7%)	†	de Moya et al. (2019)
*Physconelloides* (Ischnocera: Philopteroides)	Columbiformes	3 of 10 (30%)	†	Sweet and Johnson (2018)
*Austromenopon* (Amblycera: Meniponidae)	Aquatic birds	8 of 14 (57%)	†	Marshall (2002)
*Colpocephalum* complex (Phthiraptera: Amblycera)	Several orders of birds		†	Catanach, Valim, Weckstein, and Johnson (2018)
*Dennyus* (Amblycera: Meniponidae)	Swifts (Apodiformes)	4 of 6 (67%)	†	Page, Lee, Becher, Griffiths, and Clayton (1998)
*Dennyus* (Amblycera: Meniponidae)	Swifts (Apodiformes)	13 of 21 (57%)	†	Clayton, Al‐Tamimi, and Johnson (2003)
*Myrsidea* (Amblycera: Meniponidae)	*Catharus* sp. (Passeriformes)	No congruence		Bueter et al. (2009)
*Myrsidea nesomimi* (Amblycera: Meniponidae)	*Mimus* sp. (Passeriformes)	1 of 6 (16%)		Štefka et al. (2011)

Note

More cospeciation events or stronger phylogenetic congruence than expected by chance is indicated by a dagger (†). Number of host speciations and accompanied parasite cospeciation are indicated when available as an original publication.

Presently, studies of feather lice and their hosts are strongly biased toward temperate regions. In the tropics, however, strongly dissimilar environments and host life‐history traits may result in different patterns of host–parasite associations. There are several factors that could favor host switching in tropical environment. Higher species diversity in the tropics may increase the probability of encountering new suitable hosts. At the same time, hippoboscid flies, which are known to transfer some louse species, are typically abundant in humid tropical regions (Sweet, Chesser, & Johnson, 2017). Tropical host populations are also typically less dense and abundant than temperate zone ones (e.g., Brown, 2014) and may not represent a reliable or abundant resource. This may favor generalist parasites in the tropics which makes cospeciation less likely (Combes, 2001; Vázquez, Poulin, Krasnov, & Shenbrot, 2005). Lice may also be significantly limited by abiotic factors (Malenke, Newbold, & Clayton, 2011; Moyer, Drown, & Clayton, 2002; Rai & Lakshminarayana, 1980); hence, the high humidity and temperatures of the tropics may increase louse survival off the host, thereby facilitating host switching. Conversely, the stable conditions prevalent in the tropics (i.e., less pronounced seasonality and glacial periods), along with the higher longevity of tropical birds (Snow & Lill, 1974; Wiersma, Muñoz‐Garcia, Walker, & Williams, 2007), could result in tighter parasite–host specialization, which would decrease the success of new host colonization.

The prevailing role of host switching in the tropics for forming feather lice and bird associations is supported by the study of Weckstein (2004), who found frequent host switching between sympatric toucan species in the feather louse subspecies of *Austrophilopterus cancellosus*. Similarly, Štefka, Hoeck, Keller, and Smith (2011) found that host switching strongly influences host–parasite associations in lineages of *Myrsidea nesomimi* and their hosts, the Galápagos mockingbirds. However, analogous studies from other tropical regions, or using taxonomically broader tropical feather lice samples, are missing.

In this study, we analyze the coevolutionary processes that drive the patterns of host–parasite associations in two feather louse groups and their hosts in tropical lowland and montane forests in Cameroon (West‐Central Africa). We assess the congruence of parasite and host phylogenies and attempt to find associations that contribute to the cophylogenetic structure.

## 2 MATERIALS AND METHODS

### 2.1 Sample collection

Birds were mist‐netted and blood‐sampled at two locations in the Cameroon mountains, a pristine tropical rainforest on the south‐western slopes of Mount Cameroon (4°08′ N 9°07′ E) at elevations of 350, 700 and 2,200 m above sea level (a.s.l.) in November and December 2013 and 2014, and a highly fragmented upper montane forest situated southeast of Big Babanki village in the Bamenda Mountains (6°05′ N 10°19′ E) at elevations of 2,000 and 2,200 m a. s. l. in January and February 2016. Each bird was kept in a new paper bag before parasite collection to prevent cross‐contamination. Lice were collected from the hosts using the “fumigation chamber method” (Clayton & Drown, 2001), followed by manual inspection of the host's head plumage. Lice were stored in ethanol and subsequently classified into genera using morphological criteria (Price et al., 2003).

From the pool of parasites collected, we selected the two most diverse groups of passerine lice within our sample: lice of the genus *Myrsidea* and the *Brueelia* complex (including *Brueelia* s. str., *Guimaraesiella*, *Mirandofures* and *Sturnidoecus* sensu Bush et al. (2016) and Gustafsson and Bush (2017)), each representing one of the two feather lice suborders, that is, Amblycera and Ischnocera, respectively.

**FIGURE 1:**
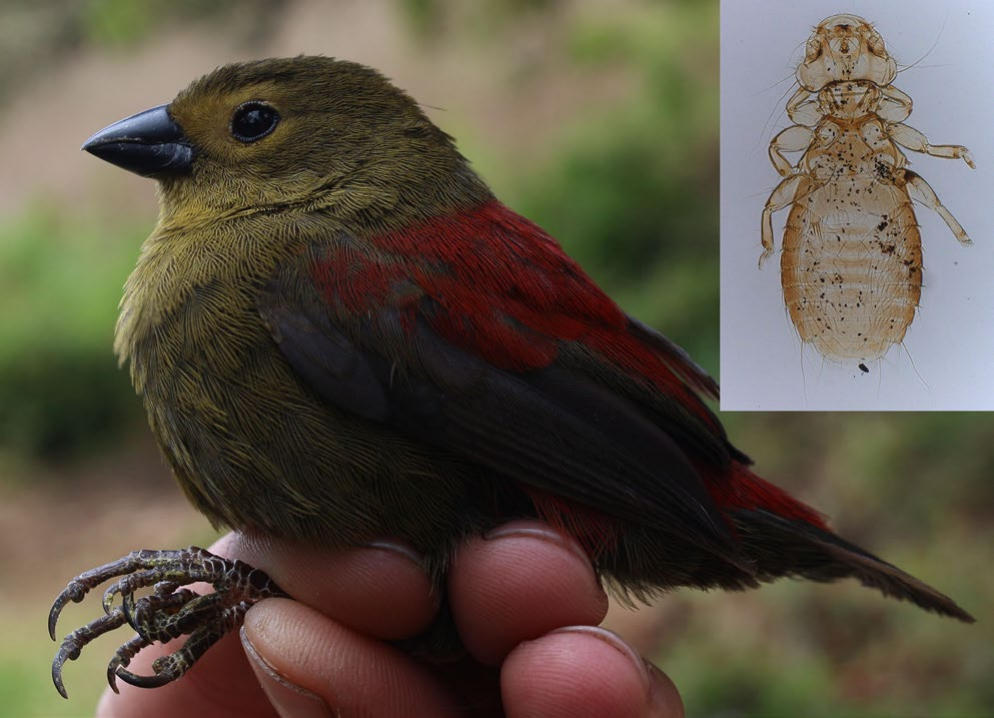
*Cryptospiza reichenovii* and its *Myrsidea* parasite

*Myrsidea* lice are host‐specific parasites found predominantly on tropical passerine species (Figure 1), though they were found also on toucans and hummingbirds (Price et al., 2003). Including more than 380 mostly neotropical described species, *Myrsidea* is one of the most specious phthirapteran genera (Kolencik et al., 2018). They seem to be intolerant to low humidity (Bush et al., 2009), feed on host feathers, and partially utilize host body fluids, including blood (Marshall, 1981).

On the contrary, lice of the *Brueelia* complex are common in both the tropics and temperate zones, and they are less host‐specific and, in addition to passerines, parasitize other bird groups, including Coraciiformes, Trogoniformes, and Piciformes (Gustafsson & Bush, 2017; Price et al., 2003). So far, over 426 species of this complex have been described (Gustafsson & Bush, 2017). Some *Brueelia* complex species are also capable of phoresis (horizontal transfer by hitchhiking) on louse flies (Hippoboscidae), which may eventually result in transport between different avian species due to the low specificity of louse flies (Keirans, 1975).

### 2.2 Molecular methods and species delimitation

Louse DNA was extracted using the Qiagen DNeasy Blood and Tissue Kit (Qiagen), following the manufacturer's protocol. To increase the DNA yield and preserve the parasite's morphological features, each louse was pierced with an entomological pin prior to incubation in proteinase K solution at 56°C for 36 hr. The exoskeleton was then removed and kept as a voucher specimen.

For species delimitation, we used partial sequences of cytochrome c oxidase subunit I (COI) of a single randomly chosen louse individual of each morphologically distinguishable group found on each infected bird. We calculated uncorrected pairwise nucleotide distances in MEGA version 7 (Kumar, Stecher, & Tamura, 2016) and utilized the web version (https://bioinfo.mnhn.fr/abi/public/abgd/abgdweb.html) of Automatic Barcode Gap Discovery (ABGD) algorithm (Puillandre, Lambert, Brouillet, & Achaz, 2011) to identify barcoding gaps in the distribution of distances. The barcoding gap separating intra‐ and interspecies distances spanned 0.03–0.17 and 0.02–0.1 in Myrsidea and the Brueelia complex, respectively. Distance matrices, histograms of pairwise nucleotide distances, and COI trees are provided in File S1–S6. According to ABGD results, we classified lice into groups characterized by intragroup COI sequence distances up to 3%. The groups were considered as unique evolutionary units and are hereafter referred to as species. A single individual of each species was used for subsequent cophylogenetic analyses. A description of new species will be given elsewhere (Sychra O., Gajdosova M., Andresova P., Albrecht T. & Munclinger P.,unpublished data).

Partial sequences of COI, wingless (wg), and 18S rDNA were sequenced in lice of both groups. In addition, partial sequences of the elongation factor 1 alpha (EF1α) and hypothetical protein EOG9X3HC5 (*hyp*) were obtained from *Myrsidea* and the *Brueelia* complex, respectively (see Table 2 for primer details). PCR conditions were identical for all loci. Amplification began with 1 min of denaturation at 94°C, followed by 35 cycles of 30 s of denaturation at 92°C, 40 s of annealing at 54°C, and 90 s of elongation at 65°C, the final step comprising 10 min of final extension at 72°C. Owing to amplification problems, we used both original and redesigned forward primers for amplification of 18S rDNA and wingless (Table 2), which resulted in slightly shorter alignments. PCR products were purified using Thermo Fisher CleanSweep™ PCR Purification Reagent (Thermo Fisher Scientific) and Sanger sequenced from both sides using the same primers as for PCR. All sequences are deposited in GenBank under accession numbers MG765475–MG765497, MK031972–MK032011, MK032012–MK032034, and MK315054–MK315114.

**TABLE 2:** Primers used for obtaining partial sequences of the elongation factor 1 alpha (EF1α) and hypothetical protein EOG9X3HC5 (hyp) in *Myrsidea* and *Brueelia* complex lice

Locus	Primer name	Primer sequence (5′–3′)	Source
COI	L6625	CCGGATCCTTYTGRTTYTTYGGNCAYCC	Hafner et al. (1994)
COI	H7005	CCGGATCCACNACRTARTANGTRTCRTG	Hafner et al. (1994)
Wingless	Lep‐wg1a	GARTGYAARTGYCAYGGYATGTCTGG	Danforth, Brady, Sipes, and Pearson (2004)
Wingless	Lep‐wg2a	ACTICGCARCACCARTGGAATGTRCA	Danforth et al. (2004)
Wingless	Wg‐Myr‐F	ATGTCTGGRTCTTGCACGGTGAARAC	This paper
18S rDNA	Ns1	GTAGTCATATGCTTGTCTC	Barker, Whiting, Johnson, and Murrell (2003)
18S rDNA	Ns2a	CGCGGCTGCTGGCACCAGACTTGC	Barker et al. (2003)
18S rDNA	Ns‐Bru‐F	TGCATGTCTCAGTGCAAGCCGAAT	This paper
hyp	BR50‐181L	CTTGARCAATTRCAGAAAAAAGC	Sweet, Allen, and Johnson (2014)
hyp	BR50‐621R	GGRTTTTCWGGAGAYCTCATCC	Sweet et al. (2014)
EF1α	EF1‐For3	GGNGACAAYGTTGGYTTCAACG	Danforth and Ji (1998)
EF1α	Cho10	ACRGCVACKGTYTGHCKCATGTC	Danforth and Ji (1998)

### 2.3 Genetic diversity and phylogenetic analysis

**FIGURE 2:**
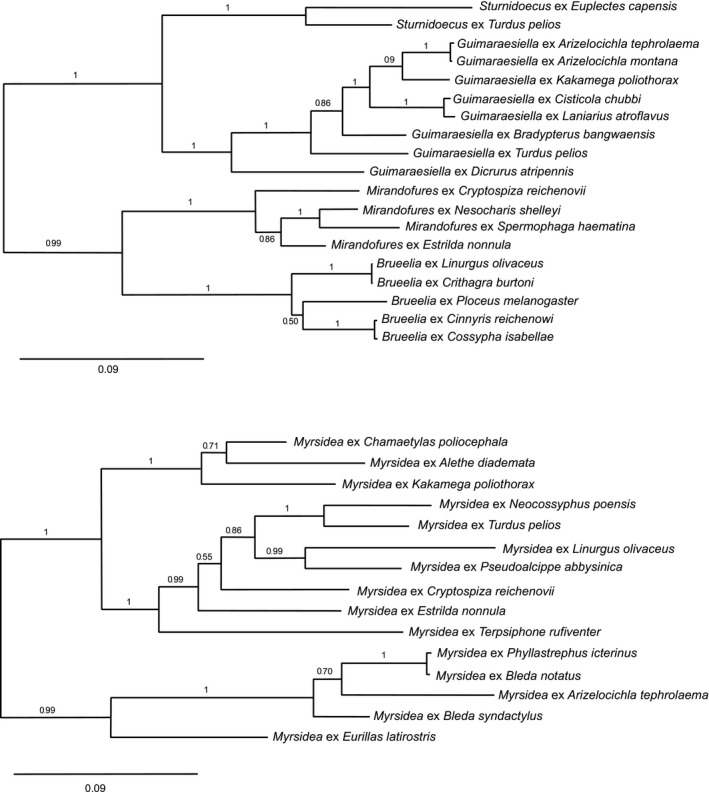
Bayesian phylogenetic trees of *Myrsidea* (based on COI, wingless, 18S rDNA, and EF1α) and the *Brueelia* complex (based on COI, wingless, 18S rDNA, and the hypothetical protein‐coding gene). Posterior probabilities are indicated at each node

Sequences of COI, wingless, 18S rDNA, and either EF1α (*Myrsidea*) or hyp (*Brueelia* complex) were aligned separately by MAFFT online version 7 (Katoh & Standley, 2013). Secondary structure of 18S rDNA was taken into consideration during alignment construction. A concatenated alignment of 1677 bp (*Myrsidea*; File S7) and 1616 bp (*Brueelia* complex; File S8) was obtained from Geneious version 7.1.9 (http://www.geneious.com; Kearse et al., 2012). Optimal genetic models for alignment subsets (each gene and each of the three codon positions of the protein‐coding genes) were assessed using PartitionFinder 1.1.1 (Lanfear, Calcott, Ho, & Guindon, 2012; Table 3). *Ricinus* sp. collected from *Platysteira laticincta* and *Philopteroides* sp. collected from *Cinnyris reichenowi* were used as out‐groups for *Myrsidea* and for the *Brueelia* complex, respectively. Bayesian analysis was conducted using MrBayes version 3.2.6 (Huelsenbeck & Ronquist, 2001; Ronquist & Huelsenbeck, 2003) using the models found by PartitionFinder for particular alignment subsets. Two independent runs were performed, each lasting 2,000,000 generations with two chains, with tree sampling every 100 generations. The first 25% of the sampled trees were discarded as burn‐in. Both runs led to consensus trees with the same topology and almost identical support values (Figure 2). Maximum‐likelihood (ML) phylogenetic approach was applied to louse molecular data using RAxML 8.2.10 (Stamatakis 2014) with GTRGAMMA model and 1,000 bootstrap replicates. Bayesian and maximum‐likelihood analyses resulted in slightly different topologies in both *Myrsidea* and the *Brueelia* complex. Hence, we utilized the Bayesian trees, which were better resolved, for cospeciation analyses and ML trees are provided only in Files (S7 and S8). Phylogenies of the avian hosts were obtained as consensus trees generated in Geneious from 2,500 trees taken from the BirdTree database (www.birdtree.org), based on Ericson et al. (2006). The trees were subsequently compared with the recent passerine phylogeny (Oliveros et al., 2019; Selvatti, Gonzaga, & de Moraes Russo, 2015) and taxonomy in the Flux (TIF) checklist, which resulted in a positional correction of *Kakamega poliothorax*.

**TABLE 3:** Models used for alignment subsets

Alignment	Model	Alignment subset
*Myrsidea*	HKY + I+ G	COI 1st position
	GTR + G	COI 2nd position
	K80 + I+G	COI 3rd position
		18S rRNA
		EF1α 3rd position
	HKY + G	Wingless 1st position
		EF1α 2nd position
	JC	EF1α 1st position
		Wingless 2nd position
		Wingless 3rd position
*Brueelia* complex	HKY + I+G	COI 1st position
	GTR + G	COI 2nd position
		hyp 2nd position
	SYM + I	COI 3rd position
		Wingless 2nd position
		Wingless 3rd position
		18S rRNA
	HKY + G	Wingless 1st position
	HKY	hyp 1st position
	HKY + G	hyp 2nd position

### 2.4 Cospeciation analysis

Cophylogenetic history was reconstructed in Jane 4 (Conow, Fielder, Ovadia, & Libeskind‐Hadas, 2010), which accepts multihost parasitism. Jane implements a reconciliation algorithm to find the most optimal scenario of cophylogenetic past. By assigning costs to events which could possibly happen during the host–parasite cophylogenetic history (e. g., cospeciation, sorting events, lineage duplication, host switching, parasite's failure to diverge), Jane finds the least costly scenario that explains the observed situation. Event costs were left as default, that is, cospeciation 0, duplication 1, duplication with host switching 2, loss 1, and failure to diverge 1. The analyses were run for 30 generations with a population size of 1,300. To test whether the reconstructed solution was better than scenarios expected by chance, we compared the cost of the reconstructed scenario with costs of 999 pseudorandom replicates generated using the “random tip mappings” approach. Tanglegrams visualizing host–parasite associations and phylogenies were created in TreeMap3 (Charleston & Robertson, 2002). Codivergence between both groups was further tested using the PACo script (Balbuena, Míguez‐Lozano, & Blasco‐Costa, 2013), using the APE (Paradis, Claude, & Strimmer, 2004) and VEGAN (Dixon, 2003) packages in R version 3.5.1 (R core Team, 2017). PACo is a specific case of Procrustean analysis, which generally assesses the level of congruence between two (or more) ordinations of multivariate data sets. More specifically, PACo is designed to test for congruence between genetic divergence of hosts and parasites. First, we calculated cophenetic distances separately for hosts and parasites based on branch lengths in corresponding phylogenetic trees. Subsequently, principal coordinate analysis (PCoA) with Cailliez correction for negative eigenvalues was applied to extract orthogonal gradients (i.e., PCoA axes) from the two distance matrices. Scores for PCoA axes were used as an input for Procrustean superimposition assessing phylogenetic codivergence between hosts and parasites. Significance of the codivergence was tested by permutations of PCoA‐scaled distances (100,000 random rearrangements with significance level being set a priori as 0.05) as described in Balbuena et al. (2013). We also extracted squared residuals from the PACo fit to assess contributions of individual host–parasite links to the final Procrustean superimposition.

As cophenetic distances were not available for *K. poliothorax* host species due to correction of its position in the tree, we omitted this species and its parasites from the PACo analysis.

## 3 RESULTS

**FIGURE 3:**
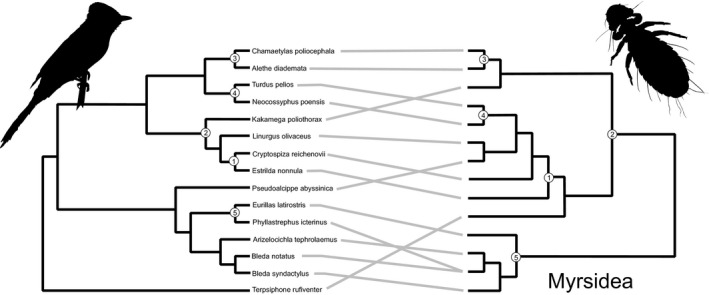
Tanglegram of passerine hosts (left) and *Myrsidea* parasites (right). The five cospeciation events found in Jane are represented by circles

**FIGURE 4:**
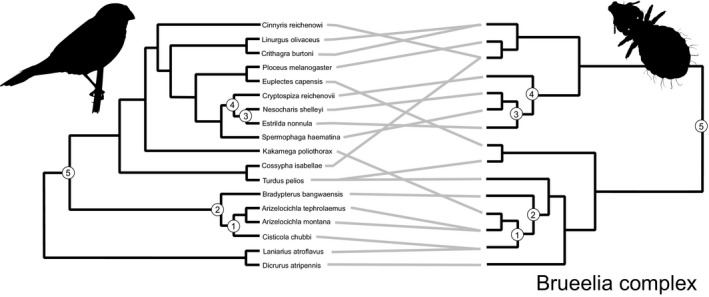
Tanglegram of passerine hosts (left) and *Brueelia* complex parasites (right). The five cospeciation events found in Jane are represented by circles

In total, 626 birds of 78 passerine species were examined for lice. Thirty‐nine birds were parasitized by *Myrsidea* lice (prevalence 6.2%) and 52 by lice of the *Brueelia* complex (prevalence 9.9%; File S12). Parasite loads were relatively low and varied between 1–38 for the *Brueelia* complex and 1–10 for *Myrsidea*. The majority of parasite species were found on a single host species; however, 1 of 14 *Myrsidea* species was found on two bird species, which involved hosts belonging to the same family (Figure 3). More cases of multihost parasites (4 of 15) were found within the *Brueelia* complex and involved associations with hosts from different families in two cases (Figure 4). One species from the *Brueelia* complex was even found on hosts of different orders, that is, the Bangwa Warbler (*Bradypterus bangwaensis* Delacour, 1943) from the Passeriformes and the Yellow‐spotted Barbet *(Buccanodon duchaillui* Cassin, 1856) from the Piciformes.

**FIGURE 5:**
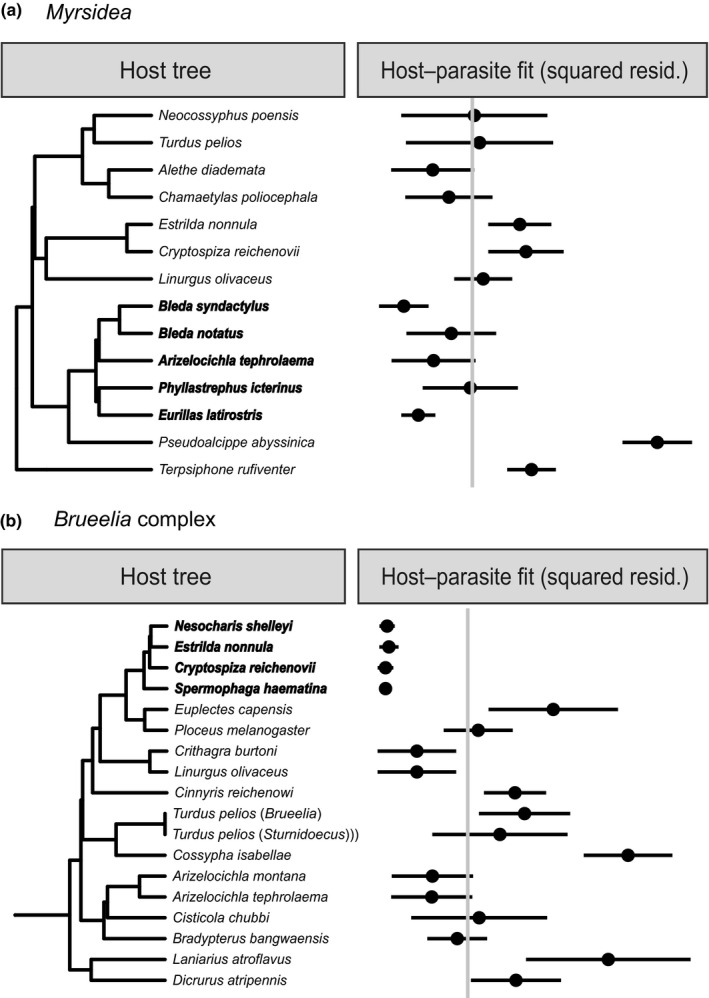
Contribution of individual host–parasite associations to the global codivergence signal based on Procrustes analysis of distance matrices between *Myrsidea* lice and their hosts (a) and *Brueelia* complex lice and their hosts (b). Squared residual 95% confidence intervals are shown. The dashed line indicates the median squared residual value. Bulbul (Pycnonotidae) host associations with *Myrsidea* lice and Waxbill (Estrildidae) host associations with *Brueelia* complex lice are shown in bold

Cophylogenetic reconstruction of *Myrsidea* revealed the most parsimonious scenario to comprise 5 cospeciation events, 0 duplications, 8 host switches, 3 sorting events, and 1 failure to speciate. More than one‐third (36%) of host speciation events were followed by parasite cospeciation (Figure 3); however, almost 9% of random solutions resulted in scenarios with the same or lower overall cost, indicating that the reconstructed solution was not significantly better than solutions created by chance. Codivergence analysis of *Myrsidea* and its hosts in PACo indicated significant congruence of host and parasite distance matrices (the goodness‐of‐fit value was 14,155.98 with *p* < .001 based on 100,000 permutations; Figure 5); however, parasites of particular host groups contributed differently to the global codivergence fit (File S11). The association of Bulbuls (Pycnonotidae) and their parasites contributed strongly to the overall congruence pattern.

The most parsimonious scenario found for the *Brueelia* complex and its hosts comprised 5 cospeciation events, 0 duplications, 9 host switches, 4 sorting events, and 4 failures to speciate (Figure 4). Hence, the frequency of parasite cospeciation (29%) appears to be slightly lower than in *Myrsidea*, though the overall cost of the scenario was significantly lower than expected by chance (i.e., Jane did not find the same or lower cost in any of 999 randomly permuted samples). There was a significant congruence between host and parasite distance matrices (the goodness‐of‐fit value was 34,205.59 with *p* < .001 based on 100,000 permutations; Figure 5), with the association between Waxbills (Estrildidae) and their parasites contributing most strongly to the overall congruence pattern (File S9).

## 4 DISCUSSION

Here, we analyze for the first time the host–parasite associations between lice and their avian hosts in the Afrotropical region. Several species of lice were detected on more than one host species; moreover, it should be noted that our sample was geographically restricted, and hence, the actual number of parasite multihost interactions may have been underestimated. The lower specificity of *Brueelia* complex lice, which were even found on phylogenetically distant hosts, can be at least partly ascribed to their ability to transfer horizontally between hosts (Keirans, 1975). We also found one *Myrsidea* species (7%) on two host species. Our study was limited to passerine hosts and lice of two model groups. Moreover, we matched only small fraction of the global diversity of the genus *Myrsidea* and the *Brueelia* complex. Deeper analyses of parasite–host interactions, preferably comparing the same groups of lice concurrently in the tropics and temperate regions, are needed to generalize our findings. However, both the multihost interactions and limited number of cospeciation events observed in this study are in good agreement with the general trend of greater parasite richness in the tropics (reviewed in Schemske, Mittelbach, Cornell, Sobel, & Roy, 2009). Under strict cospeciation scenarios, one would expect unique (one‐to‐one) parasite–host associations (Lyal, 1986). However, the number of host switches found in this study was higher than the number of cospeciation events, even though the event costs were set higher for host switching than cospeciation. Thus, our results are in agreement with previous evidence of limited cospeciation between lice and birds in other tropical regions, such as South America (Weckstein, 2004) and the Galapagos (Štefka et al., 2011).

Host switching was prevalent in the most parsimonious scenario for both the *Brueelia* complex and *Myrsidea* lice. Frequent host switching of *Brueelia* species has also been suggested in previous cospeciation analyses (Bueter, Weckstein, Johnson, Bates, & Gordon, 2009; Johnson et al., 2002) and is at least partly explained by horizontal transfer between hosts, enabled by hitchhiking of some *Brueelia* species on louse flies. However, horizontal transfer can also be mediated by other mechanisms, for example, lice may be transmitted via nest and nest‐site reuse, especially in hole nesters (Timm, 1983; Weckstein, 2004). Indeed, some of the birds in our study (*Alethe diademata*, *Chamaetylas poliocephala* and *Cossypha isabellae*) are known to be hole nesters (del Hoyo, Elliott, & Sargatal, 1997), and there is also evidence of nest and nest‐site reuse in some open nesters, for example, *Turdus pelios, Apalis pulchra,* and *Nesocharis shelleyi* (del Hoyo et al., 1997; del Hoyo, Elliott, & Sargatal, 1999, 2003). Additionally, some species (e.g., *Cinnyris reichenowi*, *Cyanomitra olivacea*, *Estrilda nonnula,* and *Spermophaga haematina*) incorporate feathers from a variety of other species into their nests (del Hoyo, Elliott, & Sargatal, 2003; del Hoyo, Sargatal, & Elliott, 2001). In this context, it should be noted that some *Brueelia* species have been shown to survive off the host for up to 200 hr (Dumbacher, 1999). Furthermore, the survival of lice during such horizontal transfers may be higher in the tropics due to increased temperature and humidity. Finally, lice may also be transmitted through direct contact between hosts in mixed‐species feeding flocks or at watering places.

The apparent incongruence between parasite and host phylogeny in *Myrsidea* lice and their hosts appears rather surprising. *Myrsidea* lice feed partially on blood (Marshall, 1981) and thus come into direct contact with the host's immune system. This may reinforce parasite coadaptation to a particular host and, as a result, lower the possibility of new host colonization. On the other hand, Clayton, Bush, and Johnson (2016) suggested limited cospeciation between lice and passerine hosts due to frequent sympatry with closely related species and the host's small body size. In the latter case, lice cannot maintain sustainable population sizes and thus face the risk of extinction. While cospeciation between passerines and their louse parasites has rarely been studied, the few analyses undertaken thus far mostly show substantial incongruence between their phylogenies (Bueter et al., 2009; Johnson et al., 2002; Štefka et al., 2011; but see Sweet et al., 2018), in accord with our own results. Further, the concept of risk of extinction on small‐bodied hosts fits well with our own findings, which suggest sorting as the prevailing event in the most parsimonious scenarios related to *Myrsidea* lice.

Despite the general incongruence between parasite and host phylogenies, PACo analysis showed a significant correlation between host and parasite phylogenetic distances, which may be at least partly interpreted through the prevalence of host switching to closely related hosts. The existence of such clade‐limited colonization has already been suggested, for example, in brood parasites of genus *Vidua* and their passerine hosts (Sorenson, Balakrishnan, & Payne, 2004) or in Monogenoidea (Platyhelminthes) and their Neotropical fish hosts (Braga, Razzolini, & Boeger, 2015). Presumably, limited phylogenetic distances between hosts also reflect sharing of host traits, which allows the parasite to utilize the same resources on a new host. As such, our results appear to be in accord with the “resource tracking hypothesis” (Timm, 1983). Nevertheless, the exact traits that facilitate host shifts remain unknown as related species tend to be similar in morphological, physiological, and behavioral features. On the other hand, congruence appeared to be higher in some host–parasite clades. Similar variation in host–parasite phylogenetic congruence has previously been recorded in *Brueelia* by Sweet et al. (2018). In our case, the congruence mainly concerned associations between *Myrsidea* lice and Bulbul (Pycnonotidae) hosts, and *Brueelia* complex lice and Waxbills (Estrildidae). Species within both these avian families are of similar size and body shape and have similar biology. They are also known to form flocks and sometimes even mixed‐species flocks. While our analysis suggested only one cospeciation event in the Bulbul clade with *Myrsidea* lice, the majority of host speciations were accompanied by parasite cospeciation in lice from the *Brueelia* complex and Waxbills. Hence, it would appear that congruence was established through different evolutionary processes in these two parasite–host association groups.

## CONFLICT OF INTEREST

The authors declare no conflict of interest.

## AUTHOR CONTRIBUTION

**Magdalena Gajdošová:** Conceptualization (equal); Data curation (equal); Formal analysis (equal); Investigation (equal); Methodology (equal); Visualization (equal); Writing‐original draft (equal); Writing‐review & editing (equal). **Oldřich Sychra:** Conceptualization (equal); Data curation (equal); Formal analysis (equal); Investigation (equal); Methodology (equal); Writing‐review & editing (equal). **Jakub Kreisinger:** Formal analysis (equal); Methodology (equal); Resources (equal); Writing‐original draft (equal); Writing‐review & editing (equal). **Ondřej Sedláček:** Investigation (equal); Resources (equal); Writing‐review & editing (equal). **Eric Djomo Nana:** Resources (equal); Writing‐review & editing (equal). **Tomáš Albrecht:** Conceptualization (equal); Funding acquisition (equal); Investigation (equal); Project administration (equal); Resources (equal); Writing‐review & editing (equal). **Pavel Munclinger:** Conceptualization (equal); Formal analysis (equal); Funding acquisition (equal); Investigation (equal); Methodology (equal); Project administration (equal); Resources (equal); Supervision (equal); Writing‐original draft (equal); Writing‐review & editing (equal).

## Supporting information

File S1–S12

## Data Availability

DNA sequence data are deposited in NCBI GenBank under accession numbers MG765475–MG765497, MK031972–MK032011, MK032012–MK032034, and MK315054–MK315114. The alignments, trees, and distance matrices are uploaded as supplements.
