# Supplementary figures and images for: Patterns of host–parasite associations in tropical lice and their passerine hosts in Cameroon
Source: Ecol Evol. 2020 Jun 18;10(13):6512–24. doi: 10.1002/ece3.6386 (PMC7381757; doi:10.1002/ece3.6386)

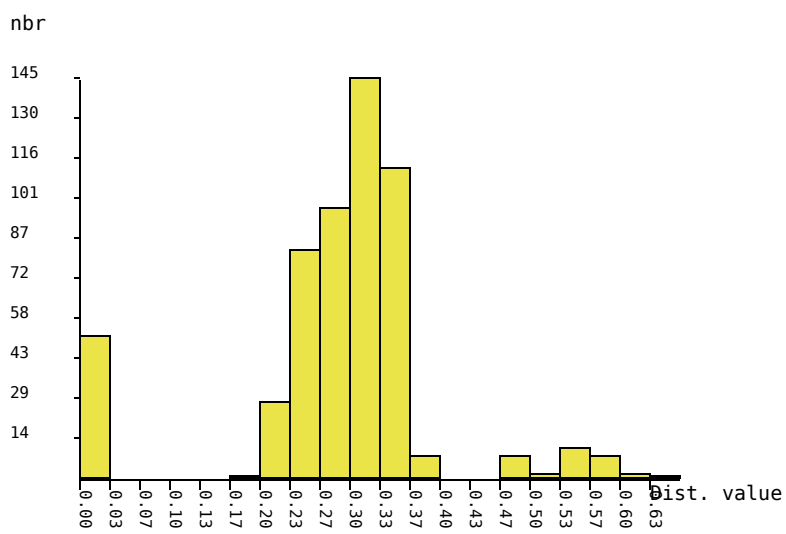

Supplement: Supplementary file 1 — File S1–S12 [file ECE3-10-6512-s001.zip › ece36386-sup-0003-FileS3.pdf]

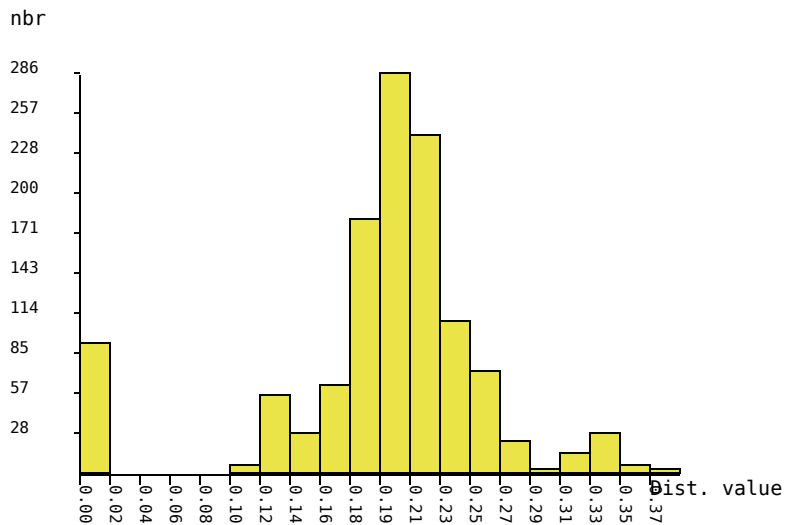

Supplement: Supplementary file 1 — File S1–S12 [file ECE3-10-6512-s001.zip › ece36386-sup-0004-FileS4.pdf]

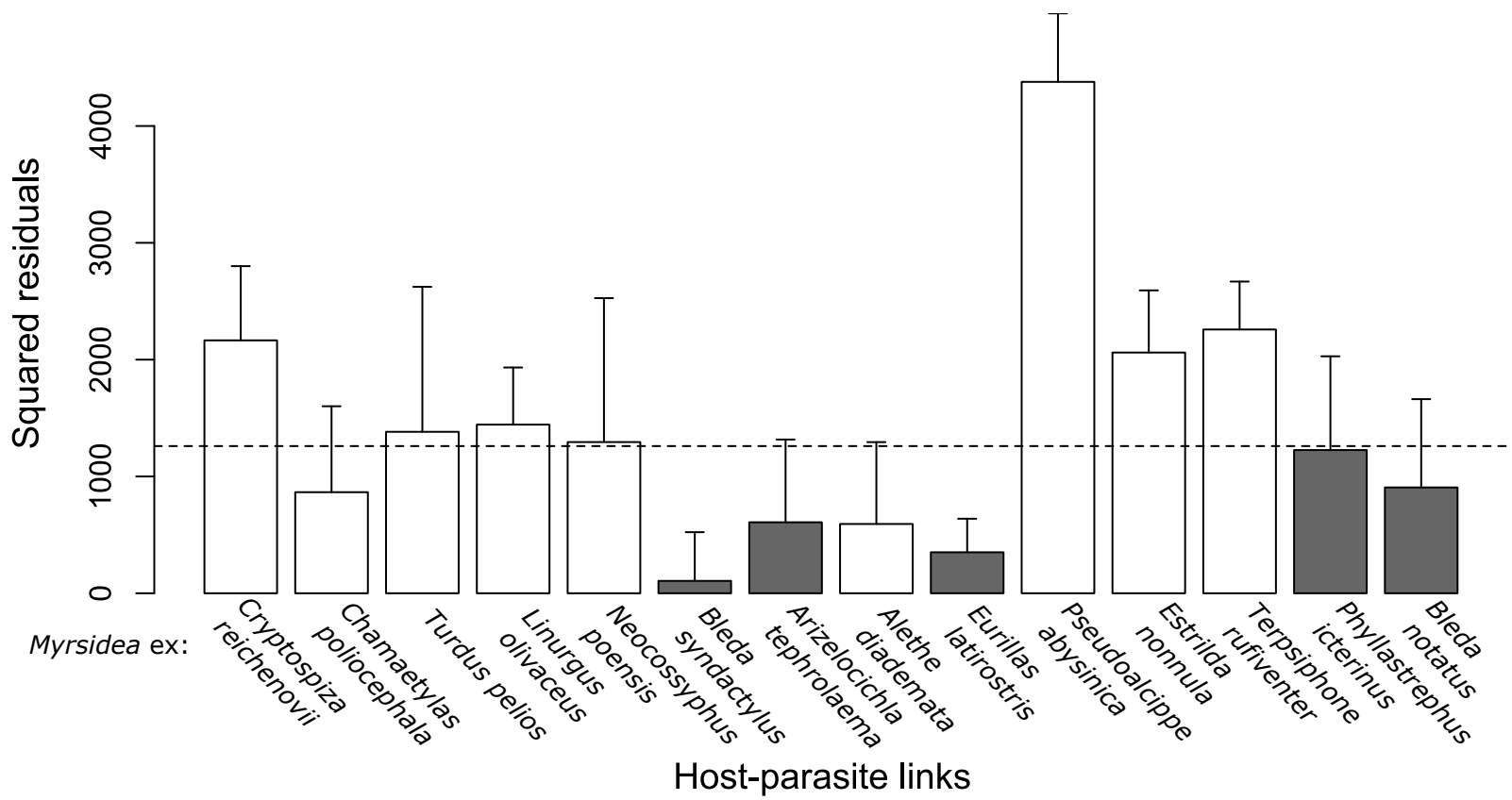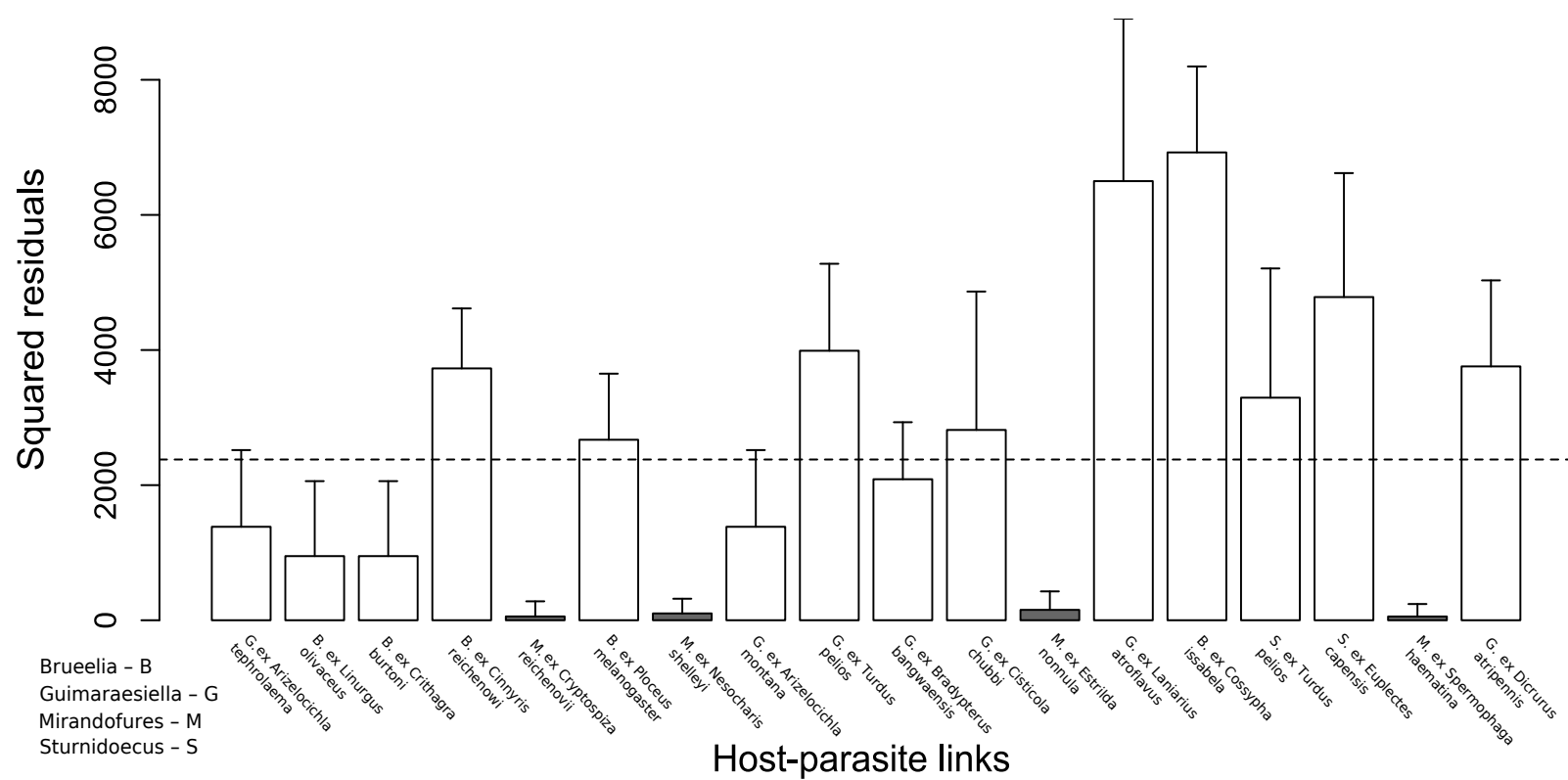

Supplement: Supplementary file 1 — File S1–S12 [file ECE3-10-6512-s001.zip › ece36386-sup-0011-FigS11.pdf]
